# A Novel Method of Fault Detection and Identification in a Tightly Coupled, INS/GNSS-Integrated System

**DOI:** 10.3390/s21092922

**Published:** 2021-04-21

**Authors:** Fan Zhang, Ye Wang, Yanbin Gao

**Affiliations:** College of Intelligent System Science and Engineering, Harbin Engineering University, Harbin 150001, China; zhangfan41@hrbeu.edu.cn (F.Z.); wangye123@hrbeu.edu.cn (Y.W.)

**Keywords:** fault detection and identification, variance shift outlier model (VSOM), INS/GNSS integrated system, tightly coupled

## Abstract

Fault detection and identification are vital for guaranteeing the precision and reliability of tightly coupled inertial navigation system (INS)/global navigation satellite system (GNSS)-integrated navigation systems. A variance shift outlier model (VSOM) was employed to detect faults in the raw pseudo-range data in this paper. The measurements were partially excluded or included in the estimation process depending on the size of the associated shift in the variance. As an objective measure, likelihood ratio and score test statistics were used to determine whether the measurements inflated variance and were deemed to be faulty. The VSOM is appealing because the down-weighting of faulty measurements with the proper weighting factors in the analysis automatically becomes part of the estimation procedure instead of deletion. A parametric bootstrap procedure for significance assessment and multiple testing to identify faults in the VSOM is proposed. The results show that VSOM was validated through field tests, and it works well when single or multiple faults exist in GNSS measurements.

## 1. Introduction

The inertial navigation system (INS) and global navigation satellite system (GNSS) compose the tightly coupled integrated navigation system directly. The fusion of the raw navigation information (pseudo-range or carrier phase measurements) of the GNSS and the inertial measurements of IMUs (inertial measurement units) is implemented in some nonlinear filters [1,2], such as the cubature Kalman filter [3] and the unscented Kalman filter [4]. An INS is a self-contained dead-reckoning system that does not rely on external information and is immune to interference. It can be assumed to be perfectly reliable. GNSS measurements are more vulnerable and are more easily jammed or interfered with. GNSS measurements may be disturbed by faults [5]. The integrated system provides superior performance when compared to either a stand-alone INS or GNSS due to their complementary characteristics [6]. Fault detection and identification play important roles in the practical applications. If a fault is not detected and identified instantly, the navigation solution will be contaminated by the fault in the measurements, and the precision and reliability will degrade. Therefore, the need for fault detection and identification in integrated navigation systems is paramount [7].

Two main types of fault detection and identification are usually employed in tightly coupled integrated navigation systems. The most common methods are fault detection and isolation (FDI), fault detection and exclusion (FDE) and fault detection and recovery (FDR). When a fault of the raw sensor signal in the integrated navigation system occurs, the FDE can provide an alarm and enable the navigation system to exclude the faulty measurements. A fault detection algorithm based on hypothesis testing in parity space was investigated by Sturza [8]. Integrity and quality control can be implemented through recursive filtering and residual chi-square tests [9]. Detection of the state chi-square relying on the residual of the current epoch can detect the abrupt changing faults, but the method does not deal with the detection of gradual changing faults well. Based on hypothesis testing, autonomous integrity monitoring by an extrapolation method was investigated for detecting gradual faults, and the measurements used in this method derive not only from current epoch but also from the previous epochs [10]. Extended receiver autonomous integrity monitoring introduced the error model of the nonlinear filter into the monitoring process [11]. Two independent detectors exist for GNSS faults and filter faults: an exclusion function can be utilized for the identification and removal of the faulty measurements, and elimination of the filter fault effect is carried out through filter recovery [12]. The former method involves an adaptive filter or a robust filter.

An adaptive filter guarantees the precision and reliability of the integrated navigation system through adaptive adjustments of the noise covariance matrix and reduction of the weights of faulty measurements, and avoids hypothesis testing. There are various kinds of adaptive estimation methods, such as the generalized maximum likelihood estimator [13], the soft-threshold optimal estimator [14] and median least squares [15]. A robust filter can reduce the weights of the fault measurements in the estimation, and its performance mainly rests on the selection of the weight matrix. Crespillo proposed robust M-estimators [16]. Compared with classical extended Kalman filters, robust M-estimators offer increased resilience at the estimator level and limit the different faulty effects in the final estimation. Appropriate thresholds are utilized for calculating the weight factor for each measurement, and adjusting the gain matrix adaptively to reduce the influence of the undetected faulty measurement. The sliding window test contributes to the improvement of the fault detection performance [17]. The filters do not need to delete the faulty measurements.

Artificial intelligence has been applied to enhance the performance of fault detection, and benefits from rapid development. A data-driven adaptive neuron fuzzy inference system for predefined faults was used for the detection of navigation sensor faults in unmanned aerial vehicles [18]. Gaussian process regression was first utilized to calculate the innovation of a Kalman filter, and improve the performance of detecting faults through the residual chi-squared test [19]. However, the heavy calculation burden of artificial intelligence on the navigation computers limits its application in integrated navigation systems.

The variance shift outlier model (VSOM) combined with an extended Kalman filter (EKF) in this paper is utilized for the detection of GNSS measurement faults and the estimation of the variance shift for each measurement, which down-weights the measurement if required. The innovation of the EKF was fitted to the VSOM used to detect and identify the faulty measurements. The VSOM can be considered as the middle ground between FDI/FDE/FDR and robust estimation [20]. Unlike the methods of FDI/FDE/FDR [11,12], the size of the variance shift in the measurement determines the partial exclusion or inclusion of the measurement in the estimation in place of the complete deletion. Compared with robust filters [16,17], the identified faulty measurements are down-weighted, but not of all the measurements are weighted. The likelihood ratio (LR) test and score test statistics were used for the detection and identification of the faulty measurements, and the parametric bootstrap procedure was implemented for the significance assessment of both the LR and score testing and for multiple testing of the statistics.

The remainder of the paper is organized as follows. In Section 2, the mathematical models of the tightly coupled, INS/GNSS-integrated navigation system and variance shift outlier model are given. The novel method based on innovation and the VSOM are proposed in Section 3. Field test results, including the static test results and dynamic test results, are shown in Section 4. The summary for this paper is presented in Section 5.

## 2. Background

### 2.1. Mathematical Model of a Tightly Coupled, INS/GNSS-Integrated Navigation System

**Figure 1:**
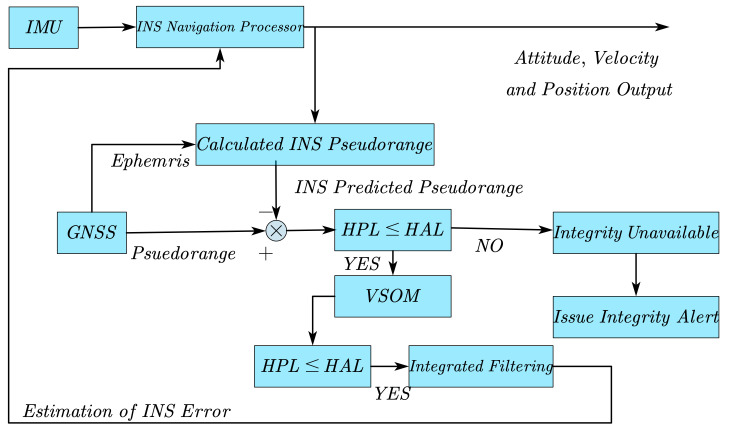
Tightly coupled, INS/GNSS-integrated navigation system architecture with a closed-loop error-state.

A tightly coupled, INS/GNSS-integrated navigation system with a closed-loop error-state, extended Kalman filter is described in this paper. The tightly coupled approach processes the raw pseudo-range GNSS measurement in a single filter, and the estimated position errors are utilized to correct the INS’s navigation solution [21]. The architecture is shown in Figure 1.

The linear dynamic equation for the error state in a time-varying linear system can be given as follows:

(1) $$\dot{x}\left(t\right)=F\left(t\right)x\left(t\right)+w\left(t\right)$$

The state vector in the filter is given by

(2) $$x={\left[{\left(\epsilon \right)}^{T},{\left(\delta v\right)}^{T},{\left(\delta r\right)}^{T},{\left(\delta \omega_{ib}^{b}\right)}^{T},{\left(\delta f^{b}\right)}^{T},{\left(\delta C_{u}\right)}^{T},\left(\delta {\dot{C}}_{u}^{T}\right)\right]}^{T}$$

where ϵ, δv and δr are the error states of the attitude, velocity and position of the INS, respectively; $\delta \omega_{ib}^{b}$ and $\delta f^{b}$ are the gyroscope and accelerometer bias vectors; $\delta C_{u}$ and $\delta {\dot{C}}_{u}$ denote, respectively, the GNSS receiver clock bias and the clock drift vector.

The linear observation equation is given by

(3) z(t)=H(t)x(t)+η(t)

Assuming the discrete-time process, the system error dynamic equation and the observation equation can be rewritten as follows:

(4) $$x_{k}=\Phi_{k|k-1}x_{k-1}+\omega_{k-1}$$

(5) $$z_{k}=H_{k}x_{k}+\eta_{k}$$

where $\Phi_{k|k-1}$, $H_{k}$ and $z_{k}$ are the transition matrix, the design matrix and the observation vector of the filter, respectively; $\omega_{k}$ is the process noise vector, which is zero-mean Gaussian white noise with a process covariance matrix $Q_{k}$; and $\eta_{k}$ is measurement noise with covariance matrix $R_{k}$, which is also zero-mean Gaussian white noise. The extended Kalman filter(EKF) algorithm consists of state updating and measurement updating. The prediction of state ${\tilde{x}}_{k}$ and its covariance matrix ${\tilde{P}}_{k}$ can be obtained by a state update as follows:

(6) $${\tilde{x}}_{k}=\Phi_{k|k-1}{\hat{x}}_{k-1}$$

(7) $${\tilde{P}}_{k}=\Phi_{k|k-1}{\hat{P}}_{k-1}\Phi_{k|k-1}^{T}+Q_{k}$$

When the observations are available, ${\hat{x}}_{k}$ is the estimation of the state vector and ${\hat{P}}_{k}$ is its covariance matrix. The measurement update in the filter is given by

(8) $${\hat{x}}_{k}={\tilde{x}}_{k}+K_{k}\left(z_{k}-H_{k}{\tilde{x}}_{k}\right)$$

(9) $${\hat{P}}_{k}=\left(I-K_{k}H_{k}\right){\tilde{P}}_{k}$$

where $K_{k}$ is the Kalman gain matrix, $r_{k}$ is the innovation vector and *I* denotes the identity matrix. The Kalman gain matrix is:

(10) $$K_{k}={\tilde{P}}_{k}H_{k}^{T}{\left(H_{k}{\tilde{P}}_{k}H_{k}^{T}+R_{k}\right)}^{-1}$$

The innovation vector is

(11) $$r_{k}=z_{k}-H_{k}{\tilde{x}}_{k}$$

When a closed loop EKF is deployed, the value of the estimated state vector feeds back to the system, and the predicted state vector ${\tilde{x}}_{k}$ becomes zero [12,21]. The innovation $r_{k}$ derives from the difference between the corrected pseudo-range vector $\rho_{GNSS}$ and the predicted pseudo-range vector derived from the solution of INS $\rho_{INS}$.

(12) $$r_{k}=z_{k}=\rho_{GNSS}-\rho_{INS}$$

Under the fault-free condition, the innovation vector $r_{k}$ is Gaussian white noise with a zero-mean, and its covariance matrix $P_{rk}$ is given as:

(13) $$P_{rk}=H_{k}{\tilde{P}}_{k}H_{k}^{T}+R_{k}$$

The horizontal alert limit (HAL) and the horizontal protection level (HPL) need to be compared with each other. When some faults in the measurements are not detected and identified, HAL and HPL could give the system protection. HAL is the maximal tolerable value of horizontal position error. When it is exceeded, an alert will be raised. The value of HAL is specified as 40 m in this paper. HPL is an upper bound. If the horizontal position error exceeds HPL, it shall be detected with a 99.9% probability. HPL needs to be computed in real time to check the integrity available and analyze the position-domain to determine whether the estimation could be used for the solution. Two computational passes are required for HPL in this tightly coupled, INS/GNSS-integrated navigation system, the first for checking integrity and the second for checking the availability of the final navigation solutions. A detailed calculation process for HPL is given in Section 3.4.

### 2.2. Variance Shift Outlier Model

The variance shift outlier model defined in this paper is the same as that in [20,22,23]. The linear model is as follows:

(14) z=Hx+η

for $z={\left(z_{1},\ldots z_{i},\ldots z_{p}\right)}^{T}$, *H* is p×q the design matrix, *x* is a q×1 parameter vector and η is a p×1 random error vector that is assumed to obey a Gaussian distribution with a zero mean and the variance $\sigma^{2}I$. The residual maximum likelihood (REML) ignoring constants LR takes the form

(15) $$\begin{array}{cc} RL & =-\frac{1}{2}\left\{\left(p-q\right)\log\sigma^{2}+\frac{{\left(z-H\hat{x}\right)}^{T}\left(z-H\hat{x}\right)}{\sigma^{2}}\right\} \end{array}$$

where $\hat{x}={\left(H^{T}H\right)}^{-1}H^{T}z$ is the best linear unbiased estimate (BLUE) of *x* under the linear model (14). The REML estimate ${\hat{\sigma }}^{2}={\left(z-H\hat{x}\right)}^{T}\left(z-H\hat{x}\right)/\left(p-q\right)$ of $\sigma^{2}$ is unbiased.

Suppose the *i*th measurement has an inflated error variance. *i* is the number less than or equal to the dimensions of *z*, which denotes the position of the fault in the measurements. The measurement has error variance $\sigma^{2}\left(\varpi_{i}+1\right)$; $\varpi_{i}\geq 0$, and $\varpi_{i}$ denotes the inflated factor in the variance of the *i*th measurement. A variance shift model for the *i*th measurement takes the form

(16) $$z=Hx+d_{i}\delta_{i}+\eta$$

where $d_{i}$ is the *i*th p×1 unit vector with 1 in the *i*th element and 0 elsewhere; $\delta_{i}$ is a random coefficient with zero-mean and its variance is $\varpi_{i}\sigma^{2}$ where $\varpi_{i}\geq 0$. $W_{i}=\varpi_{i}d_{i}d_{i}^{T}+I$ is a diagonal matrix with $\varpi_{i}+1$ corresponding to $z_{i}$ and 1 elsewhere. *I* is a p×p identity matrix. The variance matrix of *z* the data under a VSOM model (16) is

(17) $$var\left(z\right)=\sigma^{2}\left(\varpi_{i}d_{i}d_{i}^{T}\right)+\sigma^{2}I_{n}$$

The variance corresponding to the *i*th element of *z* inflates $\varpi_{i}+1$ more than the variances of the fault-free elements.

The REML log-likelihood function (REML LLF) for the *i*th VSOM (16) can be expressed as follows:

(18) $$\begin{array}{cc} RL_{i}\left(\varpi_{i},\sigma^{2};z\right) & =-\frac{1}{2}\left\{\left(p-q\right)\log\sigma^{2}+\log|W_{i}|+\log|HW_{i}^{-1}H|+\frac{{\left(z-Hx\right)}^{T}W_{i}^{-1}\left(z-Hx\right)}{\sigma^{2}}\right\}\\ \; & =-\frac{1}{2}\left\{\left(p-q\right)\log\sigma^{2}+\log|W_{i}|+\log|HW_{i}^{-1}H|+\frac{zP_{i}z}{\sigma^{2}}\right\} \end{array}$$

where $zP_{i}z={\left(z-Hx\right)}^{T}W_{i}^{-1}\left(z-Hx\right)$.

The REML LLF for the *i*th VSOM can be given in another way:

(19) $$\begin{array}{cc} RL_{i}\left(\varpi_{i},\sigma^{2};z\right)=- & \frac{1}{2}\{\log\left|H^{T}H\right|+\left(p-q-1\right)\log\sigma^{2}+\log\left[1+\varpi_{i}\left(1-c_{i}\right)\right]\sigma^{2}\\ \; & +\frac{zP_{H^{⊥}}z-s_{i}^{2}}{\sigma^{2}}+\frac{s_{i}^{2}}{\sigma^{2}\left[1+\varpi_{i}\left(1-c_{i}\right)\right]}\} \end{array}$$

where $c_{i}=d_{i}^{T}H{\left(H^{T}H\right)}^{-1}H^{T}d_{i}$, $P_{H^{⊥}}=I-H{\left(H^{T}H\right)}^{-1}H^{T}$, $s_{i}^{2}=\eta_{i}^{2}/\left(1-c_{i}\right)$, $\hat{\eta }=z-H\hat{x}$ and $\eta_{i}=d_{i}^{T}\left(z-H\hat{x}\right)$. The REML estimates of variance and inflated factor are [24]

(20) $${\hat{\sigma }}^{2}=\left\{\begin{array}{cc} \frac{\left(p-q-t_{i}^{2}\right){\hat{\sigma }}_{0}^{2}}{\left(p-q-1\right)} & t_{i}^{2}>1\\ {\hat{\sigma }}_{0}^{2} & \mathrm{otherwise} \end{array}\right.$$

and

(21) $${\hat{\varpi_{i}}}^{2}=\left\{\begin{array}{cc} \frac{\left(p-q\right)\left(t_{i}^{2}-1\right)}{\left(p-q-t_{i}^{2}\right)\left(1-c_{i}\right)} & t_{i}^{2}>1\\ 0 & \mathrm{otherwise} \end{array}\right.$$

where ${\hat{\sigma }}_{0}^{2}={\hat{\eta }}^{T}\hat{\eta }/\left(p-q\right)$ and $t_{i}^{2}=s_{i}^{2}/{\hat{\sigma }}_{0}^{2}$ are the error variance estimate and the Studentized residual with degrees of freedom p−q for the *i*th measurement under the null model (14), respectively. Since $t_{i}^{2}/\left(p-q\right)$ is distributed as beta (1/2,(p−q)/2), $t_{i}^{2}$ should less than p−q. In the case of $t_{i}^{2}=p-q$, the estimate of ${\hat{\varpi }}_{i}$ would approach infinity [25].

## 3. Methodology

A linear model that follows is used here.

(22) $$z_{k}=G_{k}x_{frame}+\eta_{k}$$

where $z_{k}$ is the innovation of the Kalman filter, which is the same as the one defined in Equation (12). $x_{frame}$ is the vector of position errors and receiver clock biases, whose dimensions are equal to three plus the number of observed constellations. $G_{k}$ is the geometry matrix defined in [21,26].

### 3.1. Establishment of Test Statistics

Normalize the measurement equation as follows (22) [21,27]:

(23) $$z_{nk}=G_{nk}x_{frame}+\eta_{nk}$$

where:

(24) $$\begin{array}{cc} P_{rk} & =P_{rk}^{1/2T}P_{rk}^{1/2}\\ z_{nk} & =P_{rk}^{-1/2}z_{k}\\ G_{nk} & =P_{rk}^{-1/2}G_{k}\\ \eta_{nk} & =P_{rk}^{-1/2}\eta_{k} \end{array}$$

$P_{rk}^{-1/2}$ is inverse of the upper triangular matrix by the Cholesky decomposition of $P_{rk}$.

Then

(25) $$\eta_{nk}∼N\left(0,I\right)$$

Equation (23) has the same form as Equation (14). According to Equations (14)–(21), some parameters under fault-free conditions can be rewritten as

(26) $$\begin{array}{cc} M & =G_{nk}{\left(G_{nk}^{T}G_{nk}\right)}^{-1}G_{nk}\\ {\hat{\eta }}_{i} & =d_{i}^{T}\left(I-M\right)z_{nk}\\ c_{i} & =d_{i}^{T}Md_{i}\\ {\hat{\sigma }}_{0}^{2} & =\frac{z_{nk}^{T}\left(I-M\right)z_{nk}}{N_{sat}-N_{sta}}\\ t_{i}^{2} & ={\hat{\eta }}_{i}^{2}/{\hat{\sigma }}_{0}^{2}\left(1-c_{i}\right) \end{array}$$

where $N_{Sat}$ is the number of observed navigation satellites which is equal to the dimensions of $z_{k}$; $N_{Sta}=N_{Const}+3$ is the dimensions of unknown parameter $x_{frame}$; and $N_{Const}$ is the number of observed constellations. ${\hat{\eta }}_{i}$ contains the inflated error variance of the *i*th satellite measurement; see Appendix A. To confirm whether there is a variance shift parameter $\varpi_{i}$ larger than zero in the *i*th measurement, testing of the hypotheses is established as follows:

(27) $$H_{0}:\varpi_{i}=0\;\mathrm{vs}.\;H_{A}:\varpi_{i}>0$$

The alternative model is fitted under the VSOM of the *i*th measurement.

#### 3.1.1. Likelihood Ratio Test Statistics

To test the hypothesis, the likelihood ratio test (LRT) statistic is established by [20,23]

(28) $$LRT_{i}=-2\left\{RL_{0}\left({\hat{\psi }}_{0};z_{nk}\right)-RL_{i}\left({\hat{\psi }}_{i};z_{nk}\right)\right\}$$

where ${\hat{\psi }}_{0}={\left(0,{\hat{\sigma }}_{0}^{2}\right)}^{T}$ is a variance estimate under the null hypothesis and ${\hat{\psi }}_{i}=\left({\hat{\varpi }}_{i},{\hat{\sigma }}_{0}^{2}\right)$ is one under the VSOM alternative hypothesis of the *i*th measurement. $RL_{0}\left({\hat{\psi }}_{0};z_{nk}\right)$ is the REML LLF for $z_{nk}$ at ${\hat{\psi }}_{0}$, and $RL_{i}\left({\hat{\psi }}_{i};z_{nk}\right)$ for $z_{nk}$ at ${\hat{\psi }}_{i}$. The subscript *i* denotes the *i*th VSOM.

Under the null hypothesis, the REML LLF could be given by

(29) $$RL_{0}\left({\hat{\psi }}_{0};z_{nk}\right)=-\frac{1}{2}\left\{\left(N_{Sat}-N_{Sta}\right)\log{\hat{\sigma }}_{0}+\log\left|G_{nk}^{T}G_{nk}\right|+\left(N_{Sat}-N_{Sta}\right)\right\}$$

and under the ith VSOM alternative hypothesis, the REML log-likelihood function is given by

(30) $$\begin{array}{cc} RL_{i}\left({\hat{\psi }}_{i};z_{nk}\right)= & -\frac{1}{2}\{\left(N_{Sat}-N_{Sta}-1\right)\log\frac{\left(N_{Sat}-N_{Sta}-t_{i}^{2}\right)}{\left(N_{Sat}-N_{Sta}-1\right)}+\left(N_{Sat}-N_{Sta}\right)\log{\hat{\sigma }}_{0}^{2}\\ \; & +\log|G_{nk}^{T}G_{nk}|+\log t_{i}^{2}+\left(N_{Sat}-N_{Sta}\right)\} \end{array}$$

Hence,

(31) $$LRT_{i}=\left\{\begin{array}{cc} \left(N_{Sat}-N_{Sta}-1\right)\log\frac{\left(N_{Sat}-N_{Sta}-1\right)}{\left(N_{Sat}-N_{Sta}-t_{i}^{2}\right)}-\log t_{i}^{2} & t_{i}^{2}>1\\ 0 & \mathrm{otherwise} \end{array}\right.$$

LRT is a monotonically increasing function of $t_{i}^{2}$.

#### 3.1.2. Score Test Statistics

There is an asymptotical equivalence between the LRT and the score test under the null hypothesis. However, there is a computational advantage to the score test statistic. The score test statistic does not need to fit the model specified under the alternative hypothesis, and it is only required to estimate under the null model [20,22]. A score test statistic calculation only requires the score vector and the information matrix under the null hypothesis. For the variance parameter $\varpi_{i}$, the score function by differentiating Equation (19) with respect to $\varpi_{i}$ is given by

(32) $$SV_{i}\left(\varpi_{i}\right)=\frac{1}{2}\left\{\frac{{\hat{\eta }}_{i}^{2}}{\sigma^{2}{\left[\left(1-c_{i}\right)\varpi_{i}+1\right]}^{2}}-\frac{\left(1-c_{i}\right)}{\left[\left(1-c_{i}\right)\varpi_{i}+1\right]}\right\}$$

Under the null hypothesis, $\varpi_{i}=0$ and $\sigma^{2}={\hat{\sigma }}^{2}$, the score function can be expressed as follows.

(33) $$SV_{i}\left(\varpi_{i}=0\right)=\frac{\left(1-c_{i}\right)\left(t_{i}^{2}-1\right)}{2}$$

The negative expected value of the second derivatives of (19) is specified as the information matrix for $\varpi_{i}$ under the *i*th VSOM, and the information matrix can be partitioned as follows:

(34) $$I=\left[\begin{array}{cc} I(\varpi_{i},\varpi_{i}) & I(\varpi_{i},\sigma^{2})\\ I(\sigma^{2},\varpi_{i}) & I(\sigma^{2},\sigma^{2}) \end{array}\right]$$

Using Equations (23)–(26), the expected information matrix under the null hypothesis is given by

(35) $$I=\left[\begin{array}{cc} \frac{{\left(1-c_{i}\right)}^{2}}{2} & \frac{\left(1-c_{i}\right)}{2{\hat{\sigma }}_{0}^{2}}\\ \frac{\left(1-c_{i}\right)}{2{\hat{\sigma }}_{0}^{2}} & \frac{\left(N_{sat}-N_{sta}\right)}{2{\hat{\sigma }}_{0}^{4}}. \end{array}\right]$$

For testing $H_{0}:\varpi_{i}=0$ against $H_{A}:\varpi_{i}>0$, the score test statistic takes the form

(36) $$S_{i}\left(\varpi_{i}=0\right)=\left\{\begin{array}{cc} SV_{i}^{2}\left(\varpi_{i}=0\right)I^{11} & U_{i}\left(\varpi_{i}\right)>0\\ 0 & \mathrm{otherwise} \end{array}\right.$$

$I^{11}$ can be obtained under null hypothesis

(37) $$I^{11}=\left\{I_{11}-I_{21}I_{22}^{-1}I_{12}\right\}$$

The score test statistic can be rewritten as

(38) $$S_{i}\left(\varpi_{i}=0\right)=\left\{\begin{array}{cc} \frac{\left(N_{Sat}-N_{Sta}\right){\left(t_{i}^{2}-1\right)}^{2}}{2(N_{Sat}-N_{Sta}-1)} & t_{i}^{2}>1\\ 0 & \mathrm{otherwise} \end{array}\right.$$

### 3.2. Significance and Multiple Testing

There are two situations in the practical scenario [23]: a measurement is suspicious and identified as a possible fault before analysis; the measurements are screened without prior information about the faulty measurements. In the second situation, ideally, all potential anomalous measurements are identified and included into the modeling process.

To determine how many and which measurements are faulty, the LR and score test statistics for each measurement are calculated. Then, testing more than one hypothesis simultaneously becomes the primary problem. For the appropriate sampling distribution of the LR and score test statistics, a parametric bootstrap method is implemented for significance assessments of the tests and multiple testing of the statistics. The parametric bootstrap procedure is can be divided into five steps:

**S1:**  Estimate (23) under the null hypothesis, obtaining parameter estimates $\hat{x}$ and ${\hat{\sigma }}^{2}$.
**S2:**  Generate new measurements
  (39) $$z_{nk}^{*}=G_{nk}\hat{x}+\eta_{nk}^{*}$$
  where $\eta_{nk}^{*}$ is simulated as $N(0,{\hat{\sigma }}^{2}I_{n})$.
**S3:**  Fit the null hypothesis (23) and obtain bootstrap LR test statistic $\left\{LRT_{i}^{*};i=1,\ldots ,N_{Sat}\right\}$ and score test statistics $\left\{S_{i}^{*};i=1,\ldots ,N_{Sat}\right\}$. Obtain the order statistics from each set.
**S4:**  Step 2 and step 3 are required to be repeated *B* times, for *B* is reasonably large. An empirical distribution(ED) of size *B* for each order statistic is generated.
**S5:**  Calculate the 100(1−α)th percentile for each order statistic for the required α, where α is the significance level.

When the LR test statistics exceed their respective thresholds (the score test is the asymptotic equivalent to LRT), these measurements are deemed to be all faulty and should fit the modified model, including a variance shift. The measurements that are deemed to be faulty will be down-weighted in the estimation procedure.

**Figure 2:**
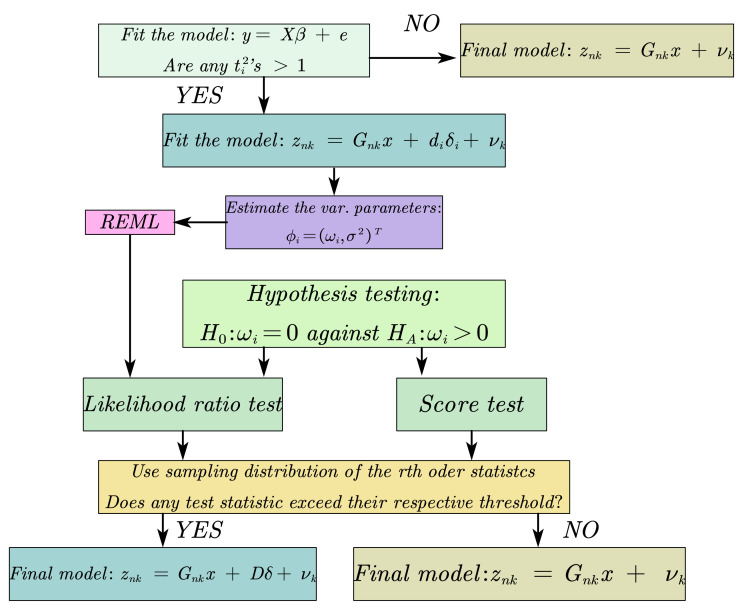
A flow chart of detecting faulty measurements without prior information about potential anomalous measurements based on VSOM.

The flow chart of detecting faulty measurements based on the VSOM approach is shown in Figure 2, where *D* is a $N_{Sat}\times N_{F}$ matrix that composes $N_{F}$ vectors with 1 in the *i*th position and 0 elsewhere, δ is a $N_{F}\times 1$ vector with random elements and $N_{F}$ is the number of faulty measurements identified.

### 3.3. Down-Weighting

After obtaining the inflated factor ϖ of the variance shift in the measurement according to Equation (21), the down-weighting process is implemented as follows:

(40) $$P_{rk}^{n}=P_{rk}^{1/2T}*W_{I}*P_{rk}^{1/2}$$

where the new covariance matrix $P_{rk}^{n}$ of the innovation is obtained by multiplying the inflated factor by $P_{rk}$, and $P_{rk}$ is defined in (13). $P_{rk}^{1/2}$ is the same as when defined in (24). $W_{i}=I+D^{T}\Pi D$; Π is a diagonal matrix with $\varpi_{i}$ in the elements corresponding to the faults, and 0 elsewhere. Then, substitute $P_{rk}^{n}$ into the Kalman filter in place of $P_{rk}$.

### 3.4. Horizontal Protection Level Computation

The computation of HPL is described as follows [28,29]. HPL1 was set to 5.33$\sigma_{H}$, where $\sigma_{H}$ was obtained from the portions of covariance matrix that corresponded to the state error vector elements about horizontal position errors, and 5.33 was derived from the missed detection rate $P_{MD}$ of $10^{-3}$/h.

The calculation of HPL2 is similar to that of traditional receiver autonomous integrity monitoring (RAIM) HPL [30]. HPL2 obtains the protection level by projecting the test statistic to the position domain. The ratio is referred to as SLOPE. The calculation of HSLOPE is given as follows:

(41) $$HSLOPE\left(i\right)=\sqrt{\left(K_{7i}^{n2}+K_{8i}^{n2}\right)/S_{ii}}$$

where $K_{7i}^{n}$ and $K_{8i}^{n}$ are the gain coefficients of biases in the measurement derived from gain matrix $K_{k}$ corresponding to the north and east directions, respectively. $S_{ii}$ is the *i*th element of diagonal matrix ${\left(I-H_{k}K_{k}\right)}^{T}\left(I-H_{k}K_{k}\right)$.

HPL2 can be calculated by

(42) $$HPL2=SLOPE_{max}P_{bias}$$

where $P_{bias}$ is the square root of the non-centrality parameter corresponding to a missed detection rate equal to $10^{-3}$/h.

HPL can be given by

(43) $$HPL=\sqrt{{\left(HPL1\right)}^{2}+{\left(HPL2\right)}^{2}}$$

## 4. Field Test

In this section, the proposed method is proven to be feasible by the a static test and a vehicular dynamic test of the tightly coupled, INS/GNSS-integrated navigation system. The fiber-optic-gyroscope-based SINS in both two experiments was independently developed by Harbin Engineering University. The sampling rate for the IMU was set at 100 Hz. The parameters of the INS are shown in Table 1. The GNSS receiver used in both two experiments was Unicore UB370. The rising edge of 1PPS was used as the trigger signal to reset the counter in the navigation computer. The counter give the raw date from IMU time information in the interval between two trigger signals. The sampling frequency of IMU was high enough, and the offset of the local system clock was not greater than 0.01 s. The output rate of receiver was set at 1 Hz. The number of bootstrap samples *B* was set at 1000. The significance level α was set at 0.01 [31].

### 4.1. Static Test

**Figure 3:**
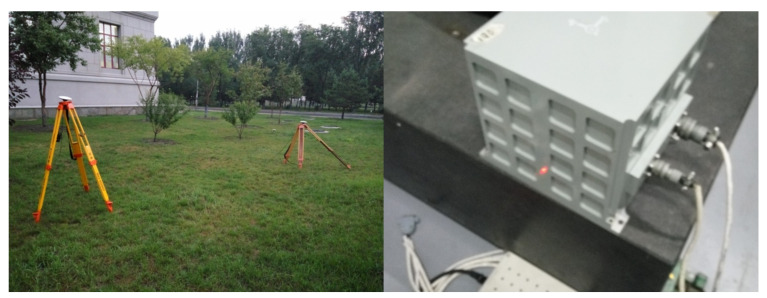
The arrangement of a tightly coupled INS/GNSS integrated navigation system for static testing.

The INS was placed on a marble isolation table, and the antenna of the receiver was placed outdoors. The GNSS receiver tracked dual-constellation navigation signals: GPS L1: 1575.42 MHz; BDS B1: 1562.098 MHz. The raw navigation data from the IMU and GNSS receiver were collected for 21,600 s. The arrangement is shown in Figure 3.

Take the data at 670 s as examples to show how VSOM detects and identifies faulty measurements. A comparison of the statistics and their respective thresholds is shown in Table 2.

Table 2 shows that the *i*th measurement is just a potential fault when $t_{i}^{2}>1$. The faults were confirmed after either an LR test or a score test. Only the eighth measurement was the fault, and the other measurements were fault-free.

**Figure 4:**
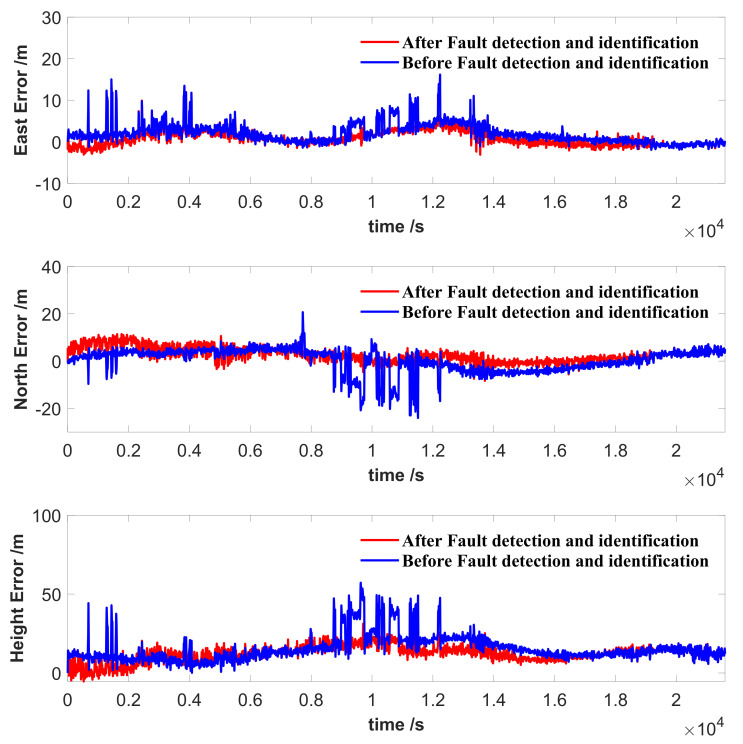
Comparison the results of the position error: before and after fault detection and identification.

The comparison of the position solutions with and without fault detection and identification based on VSOM is shown in Figure 4. The timespan of these data was six hours. There were at least four errors in the range of the 500th second to the 2000th second, obviously before fault detection and identification, and the errors caused an abrupt jump over 5 m. After the detection and identification process, the errors disappeared. The RMSE of the position before fault detection and identification was 5.8459 m, and that after fault detection and identification was 4.4656 m. Considering the position errors before fault detection and identification, this indicates that the proposed method based on the VSOM can detect and identify the faults correctly, and the faulty measurements in the estimation ensure the precision and reliability. The proposed method exhibited superior performance for the detection and identification of faults during static testing.

### 4.2. Dynamic Test

**Figure 5:**
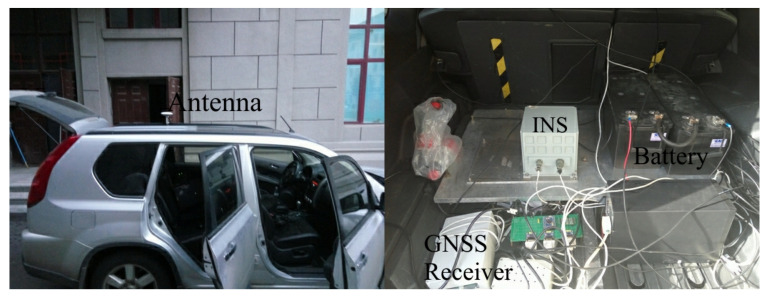
Equipment for the vehicular dynamic test.

A vehicular dynamic test was performed in an urban environment. The test equipment is shown in Figure 5. The IMU and the receiver antenna were fixed on the vehicle. The GNSS receiver tracked dual-constellation navigation signals: GPS L1: 1575.42 MHz; BDS B1: 1562.098 MHz.

**Figure 6:**
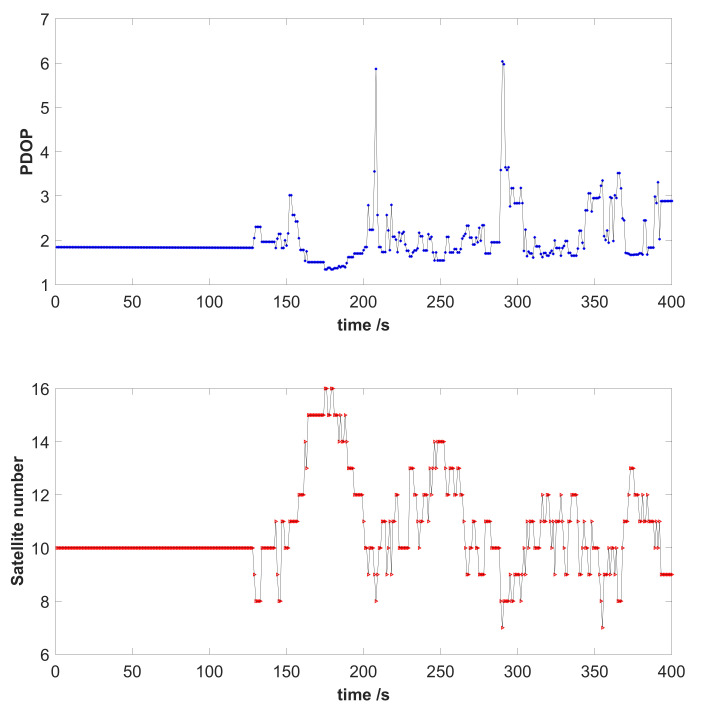
PDOP and the number of satellites during the dynamic test.

Both GPS and BDS were employed in this experiment, and the number of observed satellites was the sum of that of two systems. Figure 6 shows the positional dilution of precision (PDOP) and the number of satellites visible during the test. The average of the PDOP was 2.02, and the average number of observed satellites was 10.67.

**Figure 7:**
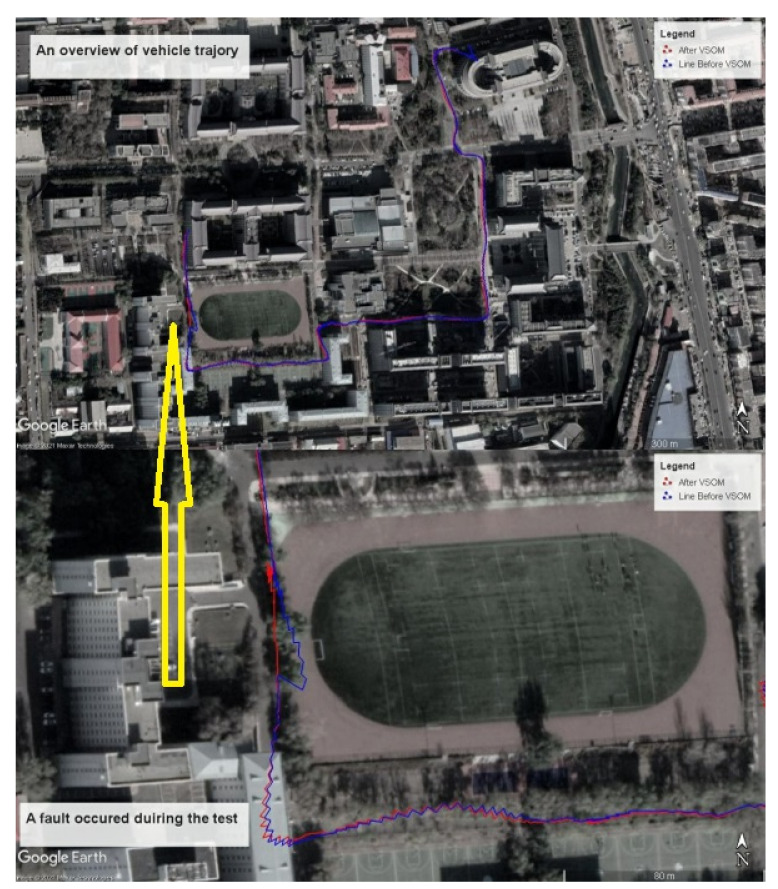
(**up**) An overview of the vehicle’s trajectory. (**down**) A fault occurred during the test.

Figure 7 shows an overview of vehicle trajectories, and shows the difference between the trajectories before and after fault detection and identification based on the VSOM. When one or more faults occurred, the trajectory became deformed. As shown in the figure below, a fault occurred at the 350th second, and the fault caused an abrupt disturbance. The proposed method was implemented, and the trajectory was not affected by the fault. Obviously, after down-weighting the fault in the estimation process, the trajectory maintained stability.

**Figure 8:**
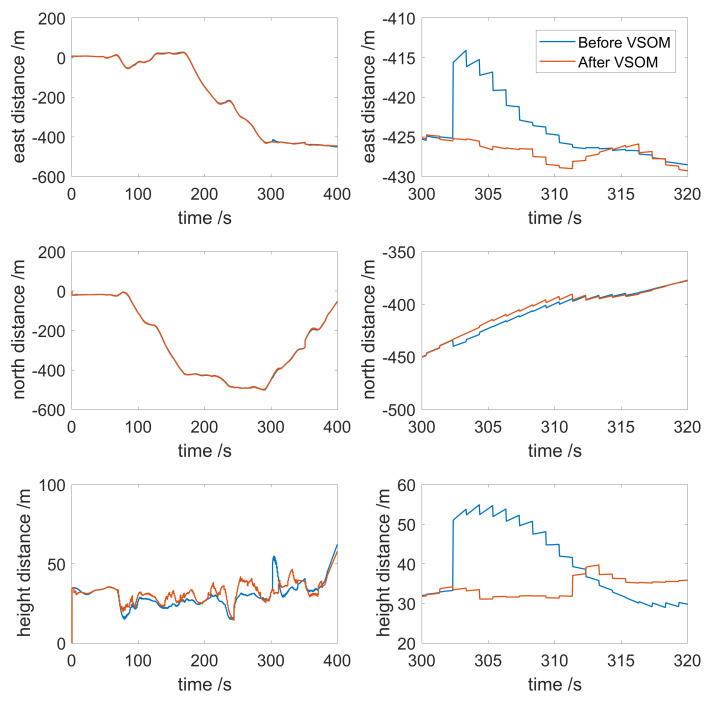
Distance from initial position to the current position. (**left**) Overview. (**right**) An in-depth look at the faults.

To further reveal the details, the distance from the initial position to the current position over time is shown in Figure 8. As shown in the figure, when the faults occurred, the east/north/upward directions all experienced some disturbance. Before fault detection and identification, the position moved 10 m to the east and 5 m to the south abruptly. A jump in the upward direction of 20 m occurred. After the fault detection and identification, the fault was down-weighted in the estimation process, and the solution was not disturbed. The transitions from the previous position to the fault position, and the fault position to the next position were seamless. This result indicates that the fault detection and identification based on the VSOM could eliminate the vibrations in the estimation caused by the faults. When the weight factors were used for down-weighting approaches to infinity, the corresponding measurements in the estimation were nearly deleted.

## 5. Conclusions

A fault detection and identification method for tightly coupled, INS/GNSS-integrated navigation systems is proposed in this paper. The method emphasizes the faults in GNSS measurements. This method is beneficial to ensuring the precision and reliability of a tightly coupled, integrated navigation system. The VSOM employed in this paper can be viewed as a trade-off between a faulty detection and exclusion algorithm and robust estimation. The size of the variance shift in the measurement is the key to partial exclusion or inclusion of the measurements in the estimation process. Either the LR statistics or score test statistics are presented as objective measures for determining whether the measurements are faulty. Parametric bootstrapping was employed in this paper to assess the significance and the handling of multiple testing. The performance of the proposed method was verified by a static field test and a dynamic field test. As shown in the results, the faults occurring during the navigation process can be detected and identified accurately. After down-weighting the fault with proper weight factors in the estimation procedure, the performance and precision can be ensured. However, the fault detection and identification based on the variance shift outlier model still need to be improved. More applicable scene tests and more flexible test criteria will be explored to perfect the method in the future.

## Figures and Tables

**Table 1:** The parameters of the INS.

	Gyroscope	Accelerometer
Bias	0.01°/h	100 μg
Bias Instability	0.01°/h	100 μg
Random Walk	$0.003{}^{\circ}/\sqrt{h}$	10 μg/$\sqrt{\mathrm{Hz}}$

**Table 2:** Values of $t_{i}^{2}$, LR and score test statistics for the fault measurements. Figures in brackets are 0.01 percentiles of the ED of *n* first-order statistics under the null hypothesis. *n* is the number of faulty measurements.

$t_{i}^{2}$	LRT	Score Test
1.6132	0.1568 (4.9503)	0.2089 (12.3899)
0.5216	0	0
0.8932	0	0
2.4981	0.7231 (4.2108)	1.2489 (10.3595)
0.0043	0	0
0.0554	0	0
0.3930	0	0
6.9031	7.7 (3.4822)	19.2598 (8.3406)
0.0985	0	0
0.8244	0	0
0.0815	0	0
0.1435	0	0
0.0027	0	0
0.9772	0	0
1.3200	0.04821 (4.3286)	0.0569 (10.6851)

## Data Availability

Not applicable.

## Appendix A

Suppose $r_{k}$ in the Equation (12) takes the form $r_{k}=z_{k}=D\delta_{GNSS}+\varepsilon_{GNSS}-G_{k}\left(b_{IC}+\varepsilon_{IC}\right)$ [12,21], where *D* is a $N_{Sat}\times N_{Sat}$ diagonal matrix with 1 in the position corresponding to the faulty measurement and 0 elsewhere; $N_{Sat}$ is defined in Equation (26); $\delta_{GNSS}$ is $N_{Sat}\times 1$, the random coefficient vector; $\varepsilon_{GNSS}$ is the random error of the GNSS measurement; $b_{IC}$ is the bias vector derived from the position of INS and the clock of the GNSS receiver; $\varepsilon_{IC}$ is the random error derived from the INS and receiver clock; and $G_{k}$ is defined in Equation (22). According to Equations (24) and (26),

(A1) $$\begin{array}{cc} \hat{\eta } & =(I-M)z_{nk}=\left(I-G_{nk}{\left(G_{nk}^{T}G_{nk}\right)}^{-1}G_{nk}\right)z_{n}k\\ & =(I-P_{rk}^{-1/2}G_{k}{\left(G_{k}^{T}P_{rk}^{-1}G_{k}\right)}^{-1}G_{k}^{T}P_{rk}^{-1/2T})P_{rk}^{-1/2}\left(D\delta_{GNSS}+\varepsilon_{GNSS}-G_{k}\left(b_{IC}+\varepsilon_{IC}\right)\right)\\ & =(I-P_{rk}^{-1/2}G_{k}{\left(G_{k}^{T}P_{rk}^{-1}G_{k}\right)}^{-1}G_{k}^{T}P_{rk}^{-1/2T})P_{rk}^{-1/2}\left(D\delta_{GNSS}+\varepsilon_{GNSS}\right)\\ \; & -\left(I-P_{rk}^{-1/2}G_{k}{\left(G_{k}^{T}P_{rk}^{-1}G_{k}\right)}^{-1}G_{k}^{T}P_{rk}^{-1/2T}\right)P_{rk}^{-1/2}G_{k}\left(b_{IC}+\varepsilon_{IC}\right) \end{array}$$

$P_{rk}$ is a positive definite matrix, so that $P_{rk}^{-1}$ is also a positive definite matrix. Then,

(A2) $$\begin{array}{cc} \; & \left(I-P_{rk}^{-1/2}G_{k}{\left(G_{k}^{T}P_{rk}^{-1}G_{k}\right)}^{-1}G_{k}^{T}P_{rk}^{-1/2T}\right)P_{rk}^{-1/2}G_{k}=0\\ \; & \hat{\eta }=\left(I-P_{rk}^{-1/2}G_{k}{\left(G_{k}^{T}P_{rk}^{-1}G_{k}\right)}^{-1}G_{k}^{T}P_{rk}^{-1/2T}\right)P_{rk}^{-1/2}\left(D\delta_{GNSS}+\varepsilon_{GNSS}\right) \end{array}$$
